# Negotiating Equitable Access to Influenza Vaccines: Global Health Diplomacy and the Controversies Surrounding Avian Influenza H5N1 and Pandemic Influenza H1N1

**DOI:** 10.1371/journal.pmed.1000247

**Published:** 2010-05-04

**Authors:** David P. Fidler

**Affiliations:** Maurer School of Law, Indiana University, Bloomington, Indiana, United States of America; London School of Hygiene & Tropical Medicine, United Kingdom

## Abstract

As part of the *PLoS Medicine* series on Global Health Diplomacy, David Fidler provides a case study of the difficult negotiations to increase equitable access to vaccines for highly pathogenic avian influenza A (H5N1) and pandemic 2009 influenza A (H1N1).

*This article is part of the* PLoS Medicine *Global Health Diplomacy series*.

## Introduction

One of the most controversial areas of global health diplomacy over the past five years has involved negotiations to increase equitable access to vaccines for highly pathogenic avian influenza A (H5N1) (HPAI-H5N1) and pandemic 2009 influenza A (H1N1) (2009-H1N1). The limited results produced by these negotiations have stimulated calls for a new global framework to improve equitable access to influenza vaccines. The prospects for such a framework are not, however, promising, because the national interests of most developed states vis-à-vis dangerous influenza strains favor retaining the existing imbalanced, reactive, and ad hoc approach to vaccine access. This article examines why negotiating equitable access to influenza vaccines in the context of HPAI-H5N1 and 2009-H1N1 has been, and promises to continue to be, a difficult diplomatic endeavor.

## Influenza Vaccine Access Controversies: HPAI-H5N1 and 2009-H1N1

The re-emergence of HPAI-H5N1 in 2004 and its spread triggered fears that the world was on the brink of a potentially devastating influenza pandemic [1]. Preparations for pandemic influenza frantically began, and included plans to develop a vaccine for a pandemic H5N1 strain. These plans ran headlong into developing-country concerns that their populations would not have access to H5N1 vaccines. These concerns, and the lack of any mechanism to ensure equitable access to vaccines and other benefits from research on influenza viruses, prompted Indonesia, in 2007, to refuse to share H5N1 virus samples with the World Health Organization (WHO) that would be used for surveillance [2],. Supported by many developing countries, Indonesia's action questioned the legitimacy of WHO's Global Influenza Surveillance Network and forced WHO and its member states to begin negotiations to create a new system of influenza virus and benefits sharing [4]. Although WHO member states agreed to establish a stockpile of H5N1 vaccine [5], the negotiations have, to date, failed to reach agreement [6].

Concerns about equitable access flared again in 2009 when a novel strain of influenza A (H1N1) emerged and spread around the world. The speed and ease with which the 2009-H1N1 strain moved meant that a vaccine was the only practical means of preventing infection, and efforts to produce a vaccine began in the late spring and early summer [7]. Developed countries placed large advance orders for 2009-H1N1 vaccine and bought virtually all the vaccine companies could manufacture [8],[9]. Developing countries and WHO identified the lack of equity in how developed countries were securing access to the vaccine [10]. WHO entered talks with manufacturers and developed-country governments to secure some vaccine for developing countries [11], and WHO and the United Nations (UN) appealed for monetary donations to purchase vaccines and other supplies to help developing countries address the 2009-H1N1 virus [12]. These efforts yielded donation pledges from manufacturers [13] and developed countries [14], but the donations still left the developing world with limited supplies [15] compared to developed countries, which would retain, even after donations, sufficient vaccine to cover their populations.

Feared and actual problems with 2009-H1N1 vaccine production, however, affected the amount and timing of vaccine available for developing countries. As of this writing, Canada had not joined other developed countries in pledging to donate vaccines, because of shortages within Canada [16], and Canada awarded its vaccine contract to a Canadian company because it feared that foreign governments might restrict exports to Canada because of vaccine shortages within their territories [17]. The Australian government made it clear to the Australian manufacturer CSL that it must fulfill the government's domestic needs before exporting vaccine to the United States [18]. The United States pledged on September 17, 2009, to donate 10% of its vaccine purchases to WHO, but on October 28, US Secretary of Health and Human Services Kathleen Sebelius stated that the United States would not donate H1N1 vaccine as promised until all at-risk Americans had access, because production problems had created shortages in the United States [19]. These fears and actions reinforced the sense that the status quo concerning equitable access to influenza vaccines for developing countries was flawed.

## Moving beyond Strain-Specific Responses: The Call for a Global Access Framework

The unsatisfactory nature of vaccine access concerning HPAI-H5N1 and 2009-H1N1 has created interest in creation of a global framework for equitable access that would become operational *before* the next influenza crisis. In a presentation to the Forum of Microbial Threats of the Institute of Medicine in September 2009, WHO's lead influenza specialist, Keiji Fukuda, described the problems experienced with the negotiations on HPAI-H5N1 virus and benefits sharing and on obtaining donations from manufacturers and developed countries for 2009-H1N1 vaccine [20]. Fukuda emphasized that the process and outcomes of the negotiations were suboptimal in terms of both public health and global equity and justice. Other experts have made similar claims concerning the moral and social justice issues at stake in equitable access to 2009-H1N1 vaccines [21],[22]. In the interests of global health and global solidarity, Fukuda argued that a framework was needed to support global responses to influenza threats and ensure equitable access to vaccines for developing countries [16]. He asserted that improving access is the central global governance issue of our times, which gives the need for a global access framework importance beyond the world of public health.

## Getting to Access: Negotiating Equitable Access to Influenza Vaccines

Negotiations to increase access to vaccines for HPAI-H5N1 and 2009-H1N1 have not proved successful for many reasons. In the Intergovernmental Meeting (IGM) on Pandemic Influenza Preparedness Framework for the Sharing of Influenza Viruses and Access to Vaccines and Other Benefits, WHO member states failed to reach agreement because they could not agree on benefit sharing [23]. Developing countries want obligatory benefit sharing in return for virus sharing, with binding terms spelled out in a Standard Material Transfer Agreement (SMTA). In contrast, developed countries want to avoid binding obligations to provide benefits (e.g., vaccines, antivirals) in exchange for access to virus samples provided by developing countries. At least one news report indicated that developed countries wanted to avoid losing their ability to place advance orders for influenza vaccine because of a binding SMTA [19].

Interestingly, the 2009-H1N1 outbreak was underway when the IGM negotiations concluded unsuccessfully, meaning that this latest influenza threat was not a “game changer” for the positions staked out by WHO member states. In fact, the manner in which the outbreak and vaccine development and use proceeded favored developed countries for two reasons. First, countries with cases of 2009-H1N1 shared virus samples with WHO for surveillance and vaccine development without a *quid pro quo* for benefit sharing. To date, Indonesia remains the only country that has refused to share virus samples; other developing countries, even those that have supported Indonesia, share their samples without requiring benefits in return. Second, developed countries were able, through advance purchase contracts, to access almost all the vaccine existing manufacturing facilities can produce [8],[9] in order to ensure they would have 2009-H1N1 vaccine for their populations—precisely the option developed countries do not want the proposed SMTA to affect.

In terms of vaccine for 2009-H1N1, donations from manufacturers and developed countries were not the product of real negotiations, given that WHO and developing countries had little leverage to influence developed countries other than rhetoric about equity, justice, and solidarity. As experts noted, the donations from manufacturers were initially made without a fixed delivery date, meaning that the donated vaccines might arrive too late to be of much benefit in developing countries [24]. Developed countries only agreed to make donations *after* (1) they learned, unexpectedly, that a one-dose regimen would immunize adults, which doubled the amount of vaccine available [25]; and (2) data from the Northern and Southern hemispheres revealed that the 2009-H1N1 virus was behaving as a mild virus and not as a killer strain [15], which reduced the threat the virus posed. In addition, developed countries pledging donations made sure that they had enough vaccine to cover their populations or, as happened with the United States, postponed donations in order to address national needs. In essence, manufacturers and developed countries incurred minimal financial, national public health, or political costs in pledging and, if necessary, delaying vaccine donations.

## Vaccine and Resource Access in International Law

What has transpired in the contexts of HPAI-H5N1 and 2009-H1N1 reflects patterns seen in other efforts to create equitable access for vaccines and drugs. Existing international legal regimes that support global health, such as the WHO Constitution, the “right to health” in human rights treaties, and the International Health Regulations 2005, do not contain specific, binding provisions on equitable access to vaccines and drugs for developing countries. WHO's interest in creating a new global framework rather than relying on existing legal agreements reinforces the lack of any specific equitable access regime. Efforts to generate equitable access are not operated through purpose-built international legal instruments, and these efforts include WHO's adoption of a nonbinding global strategy on public health, innovation, and intellectual property [26]; provision of vaccines and drugs by intergovernmental organizations (e.g., WHO, UNICEF); bilateral donation schemes (e.g., the President's Emergency Plan for AIDS Relief); and public–private and nongovernmental mechanisms that make vaccines and drugs more available to developing countries (e.g., the Global Fund to Fight AIDS, Tuberculosis, and Malaria; the GAVI Alliance; Clinton Global Initiative; Médecins Sans Frontières' Campaign for Access to Essential Medicines; the International Finance Facility for Immunization; UNITAID; and Advance Market Commitments for Vaccines).

This reality provides insight into why negotiations on virus and benefit sharing in connection with HPAI-H5N1 have, to date, failed, and why negotiations on a global access framework in the wake of the problems surrounding 2009-H1N1 would face obstacles. In short, states have not agreed to binding arrangements on more equitable access but, rather, attempt to increase such access through ad hoc, reactive, and nonbinding activities that preserve national freedom of action while demonstrating some humanitarian concern.

Moreover, the situation concerning access to vaccines and drugs reflects how states generally allocate control of and access to resources. The central principles for allocating resources in international law are (1) sovereignty for resources found within a state's territory [27], and (2) exclusive jurisdiction or control for resources found seawards from coastal states (e.g., the Exclusive Economic Zone in the law of the sea) [28]. International relations provide few, if any, examples of states establishing a global framework to allocate resources, or the benefits derived from their exploitation, equitably. The most famous effort occurred in the negotiation of the UN Convention on the Law of the Sea (UNCLOS) in the 1970s and early 1980s and involved designating mineral resources found beyond 200 nautical miles from coastal states as the “common heritage of mankind,” which would be exploited under jurisdiction of an International Seabed Authority, with benefits accruing to developing countries [29]. However, the United States and other developed countries opposed this aspect of UNCLOS, which, because of this opposition, has been revised to reflect what these developed countries prefer concerning exploitation of these mineral resources [30].

The problems of equitable access to vaccines and drugs reflect these larger patterns in international law and international relations. As Indonesia's assertion of “viral sovereignty” demonstrates, states have sovereignty over biological samples isolated within their territories. Negotiations within the WHO [5] and the IGM [19] have re-emphasized that states have sovereignty over biological resources found within their jurisdictions. Similarly, states in which vaccines and drugs are manufactured have sovereignty over the manufacturing process and the products themselves, until they are exported. States that import vaccines and drugs then have sovereignty over such resources and, absent a binding obligation, may allocate them however they wish. Negotiations to create a global access framework that more equitably distributes influenza vaccines would need to navigate through triple claims of sovereignty—a very tall order, without even factoring in the divergence of national interests seen in the IGM negotiations on virus and benefit sharing and the access problems associated with vaccine for 2009-H1N1.

## Conclusion

Increasing equitable access to vaccines for dangerous influenza strains represents a difficult challenge for global health diplomacy, a challenge this article has addressed in only a preliminary manner. Efforts to recalibrate virus- and benefit-sharing in connection with HPAI-H5N1 through intergovernmental negotiations have not, so far, been successful. The manner in which access to vaccine for 2009-H1N1 played out highlights why the interests of developed and developing countries diverge in this context, and the reasons behind this divergence deserve deeper study. Existing international legal regimes on global health provide no templates for negotiating the new global access framework that WHO and others perceive is necessary. Similarly, negotiations for equitable access to resources, or the benefits of their exploitation, have generally failed in other areas of international relations, dimming prospects that precedents for a global access framework for pandemic influenza vaccines can be found outside the global health context. The default rules for allocating resources in international law rely on the principle of sovereignty, and these rules hold in the context of virus samples and vaccine supplies, as demonstrated with HPAI-H5N1 and 2009-H1N1.

Even the emergence of the first pandemic strain of influenza in 40 years in 2009 did not break the pattern of state behavior with respect to equitable access to a valuable but scarce resource. The appearance of a more severe influenza strain will reinforce rather than overcome this pattern, because developed countries will prize their power and flexibility of action more in a severe pandemic than in a mild one, thus making hope for a crisis-sparked breakthrough misguided. The negotiating path that could lead to a new global access framework for influenza vaccines is not apparent, especially in a context in which aggregate global production capacity is woefully inadequate, the geographic location of production facilities is concentrated in developed countries, timelines for developing new vaccines create problems for rapid prevention strategies, and existing manufacturing technologies and distribution systems require improvements.

The need to increase global production capacity, diversify locales for manufacturing facilities, decrease the time from “lab to jab,” and reduce production and distribution uncertainties, has been recognized for years without sufficient progress being made, as evidenced by the HPAI-H5N1 and 2009-H1N1 controversies. Further research is required on ways in which states and non-state actors can address these problems through negotiated collective action. The diplomatic environment may have been made more difficult by accusations made and hearings held by officials in the Council of Europe that WHO succumbed to pressure from the pharmaceutical industry to declare a “false pandemic” and support development and use of a vaccine [31],[32]. In the environment that exists on these issues, diplomatic advances will not be made simply by repeated claims that an undefined “global framework” is required because more equitable access is the just and moral end all states should seek.

## Abbreviations

2009-H1N1: pandemic 2009 influenza A (H1N1)
HPAI-H5N1: highly pathogenic avian influenza A (H5N1)
IGM: Intergovernmental Meeting
SMTA: Standard Material Transfer Agreement
UN: United Nations
UNCLOS: UN Convention on the Law of the Sea
WHO: World Health Organization
